# Identification of B‐cell dominant epitopes in the recombinant protein P29 from *Echinococcus granulosus*

**DOI:** 10.1002/iid3.611

**Published:** 2022-04-19

**Authors:** Yongxue Lv, Shasha Li, Tingrui Zhang, Yazhou Zhu, Jia Tao, Jihui Yang, Liangliang Chang, Changyou Wu, Wei Zhao

**Affiliations:** ^1^ School of Basic Medicine Ningxia Medical University Yinchuan China; ^2^ Ningxia Key Laboratory of Prevention and Control of Common Infectious Diseases Ningxia Hui Autonomous Region Yinchuan China; ^3^ Institute of Immunology, Zhongshan School of Medicine Sun Yat‐sen University Guangzhou China

**Keywords:** B‐cell epitope, *Echinococcus granulosus*, *rEg.P29*

## Abstract

**Introduction:**

*Echinococcus granulosus* (*E*. *granulosus*) causes a hazardous zoonotic parasitic disease. This parasite can occupy the liver and several areas of the body, causing incurable damage. Our previous studies have provided evidence that the recombinant protein P29 (*rEg.P29*) exhibit immune protection in sheep and mice against pathological damage induced by *E. granulosus*, showing its potential as candidate for vaccine development. However, information on the B‐cell epitopes of *rEg.P29* has not yet been reported.

**Methods:**

Immunological model was established in mice with *rEg.P29*. SDS‐PAGE and Western blot were used to identify protein. Screening for B‐cell dominant epitope peptides of *rEg.P29* by enzyme‐linked immunosorbent assay (ELISA) and immune serum. Dominant epitopes were validated using ELISA and flow cytometry. Multiple sequence alignment analysis was performed using BLAST and UniProt.

**Results:**

Immunization with *rEg.P29* induced intense and persistent antibody responses, and the epitope of the dominant antigen of B cells are identified as *rEg.P29*_166–185_ (LKNAKTAEQKAKWEAEVRKD). Anti‐*rEg.P29*_166–185_‐specific antibodies lack epitopes against IgA, IgE, and IgG3, compared to anti‐*rEg.P29*‐specific antibodies. However, anti‐*rEg.P29*_166–185_ IgG showed comparatively higher titers, as determined among those peptides by endpoint titration. In addition, *rEg.P29* and *rEg.P29*_166–185_ promote B‐cell activation and proliferation in vitro. The dominant epitopes are relatively conserved in different subtypes of *the rEg.P29* sequence.

**Conclusion:**

*rEg.P29*_166–185_ can act as a dominant B‐cell epitope for *rEg.P29* and promote cell activation and proliferation in the same way as *rEg.P29*.

## Graphical abstract

Our study provides information for the study of *rEg.P29* peptide vaccines and sheds light on the rational design of vaccines.

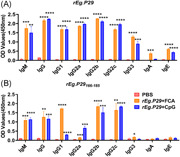

## 1 INTRODUCTION

*Echinococcus granulosus* is a zoonotic parasitic disease with an extremely serious economic burden and poses great danger in human health.^1^ Western China has a high incidence of hydatid disease.^2^ *E. granulosus* grows slowly, and more than 80% of echinococcosis cysts occur in the host liver, causing incurable damage.^3^, ^4^ Currently, the main treatment methods for patients with cystic echinococcosis (CE) are surgery and drug treatment^5^; however, drugs generally have side effects. Meanwhile, surgical treatment has a high recurrence rate and results in huge economic pressure and body damage to patients.^6^, ^7^, ^8^

The massive use of anthelmintics has caused a series of problems, such as drug resistance, drug residues, and environmental pollution.^3^ Vaccines are safe and residue‐free and are important tools for disease prevention and control.^9^, ^10^ However, traditional vaccines are composed of attenuated or inactivated pathogenic microorganisms and may cause unwanted or harmful immune responses in the body.^11^ Therefore, the development of an effective and safe vaccine against parasitic diseases is important in animal husbandry and public health.

Despite the wide range of preventative approaches explored, a human vaccine against *E. granulosus* is not yet available. In fact, the *E. granulosus* vaccine has been proposed for a long time, and many candidate proteins have been studied, such as *Eg95*, antigen B, and *rEg.P29*.^12^, ^13^, ^14^, ^15^, ^16^ Our previous study found that *rEg.P29* showed 94.5% and 96.6% protective efficacy in sheep and mouse models with secondary infection, respectively, and induced strong cellular and humoral immune responses against *E*. *granulosus* infection.^16^, ^17^ However, the anti‐infective mechanism of *rEg.P29* is still unclear.

In recent years, as our understanding of the immune response has improved and research on vaccine production has become more refined, the search for the most precise vaccine components, that is, antigenic epitopes, in recombinant vaccines has begun. These antigenic epitopes represent the smallest immunogenic regions of protein antigens and can induce a specific immune response with the desired effects in the body.^18^ Considering the importance of *rEg.P29*, an epitope vaccine containing moderate antigenic peptides of the gene may serve as an efficient vaccine against *E. granulosus* infection.

Studies have shown that B‐cell‐mediated humoral immune responses play an important role against diseases.^18^ Based on this study, we sought to screen for a dominant B‐cell epitope of *rEg.P29* and provide a basis for the construction of peptide‐based Vaccines for *rEg.P29*.

## 2 MATERIALS AND METHODS

### 2.1 Animals and immunizations

**Figure 1:**
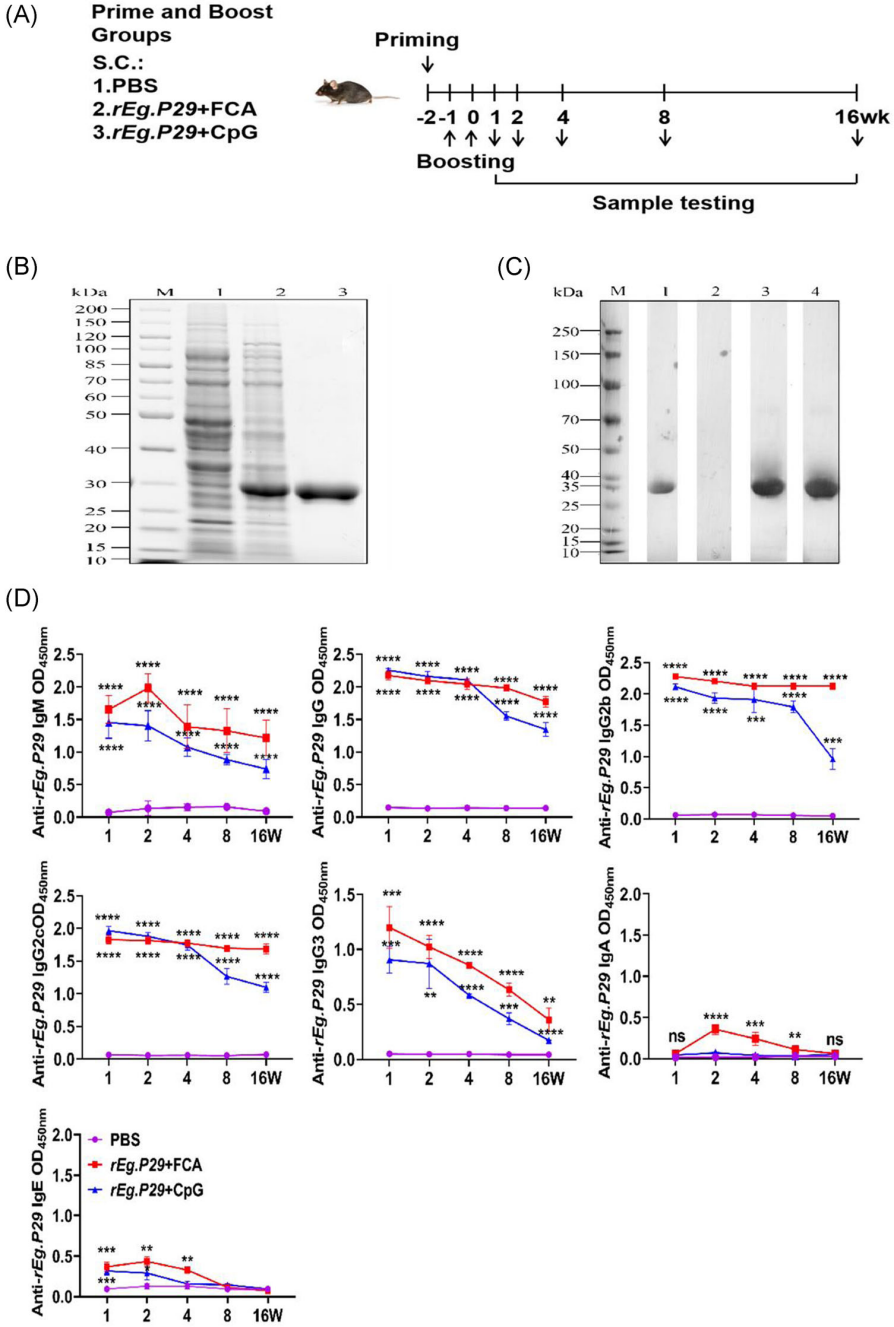
*rEg.P29* immunization induced a strong humoral immune response. Mice were primed and boosted with PBS, *rEg.P29*+FCA, or *rEg.P29*+CpG, following the prime‐boost protocol. (A) At the indicated time interval, mice were killed and serum was separated by centrifugation. (B) The expressed and purified recombinant P29 protein was detected by SDS‐PAGE analysis. Lane M, protein marker with molecular mass indicated on the left; Lane 1, *Escherichia coli* containing pET28a before IPTG induction; Lane 2, *E. coli* containing pET28a‐P29 6 h after induction; Lane 3, purified *rEg.P29* using His‐affinity chromatography, as indicated by the arrow. (C) Western blot identifies *rEg.P29*. Purified *rEg.P29* was immunoblotted with anti‐His tag antibody or postimmunized serum from mice. M, protein marker; Lane 1, anti‐his antibody; Lane 2, serum in PBS group; Lane 3, immune serum in *rEg.P29*+CpG group; Lane 4, immune serum in *rEg.P29*+FCA group. Anti‐*rEg.P29* specific antibodies were detected by ELISA. (D) ELISA plate was coated with *rEg.P29* (10 μg/ml). Then, serum levels of anti‐*rEg.P29* antibodies were measured by ELISA using HRP‐labeled anti‐mouse antibodies. The absorbance was read at 450 nm. ****p < .0001, ***p < .001, **p < .01, ns, not significant, *p* > .05

This study was approved by the Experimental Animal Ethics Committee of the Ningxia Medical University. C57BL/6 female mice (SCXK2016‐0006) aged 6–8 weeks were purchased from the Animal Center of Ningxia Medical University and kept for acclimatized feeding for 7 days before the experiment. Immunization protocols per mouse are summarized briefly as follows: 20 μg *rEg.P29* was mixed with 20 μg CpG ODN 1826 (*rEg.P29* + CpG), or 50 μl Freund's complete adjuvant (*rEg.P29* + FCA), and phosphate‐buffered saline (PBS) was used as control. Booster immunization was performed using Freund's incomplete adjuvant. For subcutaneous immunization, the mixture was suspended in PBS and injected in the lower quadrant of the abdomen (100 μl/mouse). Immunization was performed three times, 1 week apart (Figure 1A).

### 2.2 Amino acid sequence

The complete amino acid sequence of *rEg.P29* was obtained from GenBank (accession number XP_024351425.1).

### 2.3 Antigen and adjuvant

Protein purification and expression were performed as described previously.^15^ Briefly, the positive strain was induced overnight with 0.05 mg/ml isopropyl‐b‐d‐thiogalactoside (IPTG; Invitrogen) at 37°C to express the recombinant protein P29, which was then purified using an anti‐His‐tagged nickel purification column (Merck). Purified *rEg.P29* was identified using Western blot analysis. A BCA Kit (KeyGEN Biotech Products) was used to detect the protein concentration. Next, an overlapping peptide library of *rEg.P29* and CpG ODN 1826 (TCCATGACGTTCCTGACGTT) was synthesized with98% purity with the assistance of Shanghai Shenggong Biological Co., Ltd. Complete and incomplete Freund's adjuvants were purchased from Sigma‐Aldrich.

### 2.4 Sample collection and cell preparation

Blood samples were obtained from the orbit, and the serum was collected and purified via centrifugation at 400 × *g* at 4°C for 10 min. Splenocytes were isolated from the tissue by filtering the tissue through a 70‐μm strainer in a sample diluent, and the prepared cell suspension was transferred to a density gradient (Tianjin Haoyang biological products). Spleen lymphocytes were separated via centrifugation at 450×*g* for 20 min.

### 2.5 Enzyme‐linked immunosorbent assay for antibody production

Enzyme‐linked immunosorbent assay (ELISA) plates were coated with individual peptides or *rEg.P29* at 10 μg/ml and incubated overnight at 4°C. The plates were washed five times with 0.05% Tween 20 PBS (PBST) and blocked with 5% skim milk powder in PBST at 37°C for 1 h. After washing five times with PBST, the plates were incubated with primary serum (1:100) in PBS (with 10% FBS) for 2 h and washed five times with PBST for 2–3 min. One hundred microliters each of horseradish peroxidase (HRP)‐conjugated anti‐mouse IgM, IgG, IgA, IgG1, IgG2a, IgG2b, IgG2c, and IgG3 (Cat nos.: ab97230, ab97023, ab97235, ab97240, ab97245, ab97250, ab97255, and ab97260, respectively; Abcam), and IgE (Cat no.: PA1‐84764; Invitrogen) were added enzyme plates and incubated at 37°C for 1 h. After washing, 3,3′,5,5′‐Tetramethylbenzidine (TMB) was added for 8–10 min, and the reaction was stopped by 2 M H_2_SO_4_. The absorbance was measured at 450 nm within 15 min using an ELISA reader (Thermo Fisher Scientific).

### 2.6 Flow cytometry analysis

For the flow cytometry assay, phycoerythrin‐Texas Red (PE‐CF594) conjugated‐CD3, allophycocyanin‐Cyanin 7 (APC‐Cy7) conjugated‐CD19, phycoerythrin (PE) conjugated‐CD25, and Alexa Fluor700 conjugated‐CD69 were purchased from BD Biosciences, whereas Fixable Viability Dye eFluor™ 506 was obtained from Invitrogen.

Splenic lymphocytes were suspended in complete RPMI 1640 medium (HyClone) supplemented with 10% heat‐inactivated fetal calf serum (GeminiBio), 100 μg/ml streptomycin, 100 U/ml penicillin, 2 mM l‐glutamine, and 50 μM 2‐mercaptoethanol (Gibco). B‐cell activation was detected using flow cytometry. Briefly, cells were incubated with or without *rEg.P29* or peptide (15 μg/ml) at 37°C, 5% CO_2_ for 24 and 72 h. The cells were washed two times with PBS containing 0.1% bovine serum albumin (BSA) and 0.05% sodium azide (Buffer1). Then, the cells were stained with monoclonal antibodies (CD3, CD19, CD25, and CD69) for 30 min at 4°C in the dark. After washing with Buffer1, the cells were suspended in 100 µl of PBS and subjected to FACSCelesta (BD) for analysis and data collection.

As for proliferation, the cells were washed two times with pre‐warmed PBS supplemented with 0.1% BSA, carboxyfluorescein diacetate succinimidyl ester at a final concentration of 2.5 µmol/L (CFSE, Invitrogen) was added, and the resulting mixture was incubated for 15 min at 37°C in the dark. The reaction was terminated with precooled RPMI1640 containing 10% FBS, incubated at 4°C for 5 min, and washed two times with precooled RPMI1640 containing 10% FBS. CFSE‐labeled cells were then cultured with or without 15 µg/ml *rEg.P29* or peptide for 3 and 5 days at 37°C, 5% CO_2_. Cell samples were collected and stained with fluorochrome‐conjugated mAbs for phenotyping at 4°C in the dark. The samples were subjected to FACSCelesta™, and data were analyzed using FlowJo software (TreeStar).

### 2.7 Multiple sequence alignment

The amino acid sequences of P29 of different subtypes of *E. granulosus* were obtained using BLAST, and the conserved dominant epitopes were analyzed using UniProt (https://www.uniprot.org/). Amino acid sequences obtained from *E. granulosus* were as follows: AHA85389.1 (*E. granulosus s.s*. [G1]), AHA85390.1 (*E. granulosus s.s*. [G1]), AHA85391.1 (*E. equinus*), AHA85392.1 (*E. ortleppi*), AHA85393.1 (*E. canadensis* [G6]), AHA85394.1 (*E. canadensis* [G7]), AHA85395.1 (*E. canadensis* [G10]), AHA85396.1 (*E. multilocularis*), AHA85397.1 (*E. multilocularis*), AHA85398.1 (*E. multilocularis*), and AHA85399.1 (*E. multilocularis*).

### 2.8 Statistical analysis

All statistical analyses were performed using GraphPad Prism 8.0 (GraphPad Software Inc.) and SPSS 22.0. Unpaired Student's *t*‐test was used for comparisons between two groups, and one‐way or two‐way analysis of variance was used for comparisons among more than two groups. The least significant difference test or Student–Newman–Keuls test was used to analyze data with normal distribution and homogeneous variance. Dunnett's T3 test or independent sample *t*‐test was used to analyze data with normal distribution but had uneven variance. Data are represented as mean or mean ± SD. ****p < .001; ***p < .005; **p < .01; *p < .05 were considered statistically significant, whereas *p* > .05 was considered not significant (ns), as stated in figure legends.

## 3 RESULTS

### 3.1 Expression, purification, and identification of *rEg.P29*

The *rEg.P29* was successfully expressed and purified. SDS‐PAGE analysis showed that *rEg.P29* had a molecular weight of 31 kDa (Figure 1B). The protein concentration was 1 mg/ml, and the protein was stored at −80°C). Western blot analysis showed that *rEg.P29* could not be recognized by antibodies in the mouse serum samples from the PBS group but could be recognized by a mouse monoclonal His‐tag antibody and mouse sera from the immunization group (Figure 1C).

### 3.2 *rEg.P29* induced a sustained antibody response

To assess whether *rEg.P29* induces specific immune responses in mice, we studied the antibody responses to *rEg.P29* immunization in mice sera. Both immunization groups were compared with the control group. Immunization with *rEg.P29* induced a high level of anti‐*rEg.P29‐*specific antibodies. The antibody responses peaked at Week 2 after boost immunization and were maintained for more than 16 weeks, except for IgA and IgE. Specifically, IgA and IgE levels gradually decreased at Week 4, but IgG remained at high levels. Both immunization groups were compared with the control group (Figure 1D).

### 3.3 Immunization induced a polarized Th1 response

**Figure 2:**
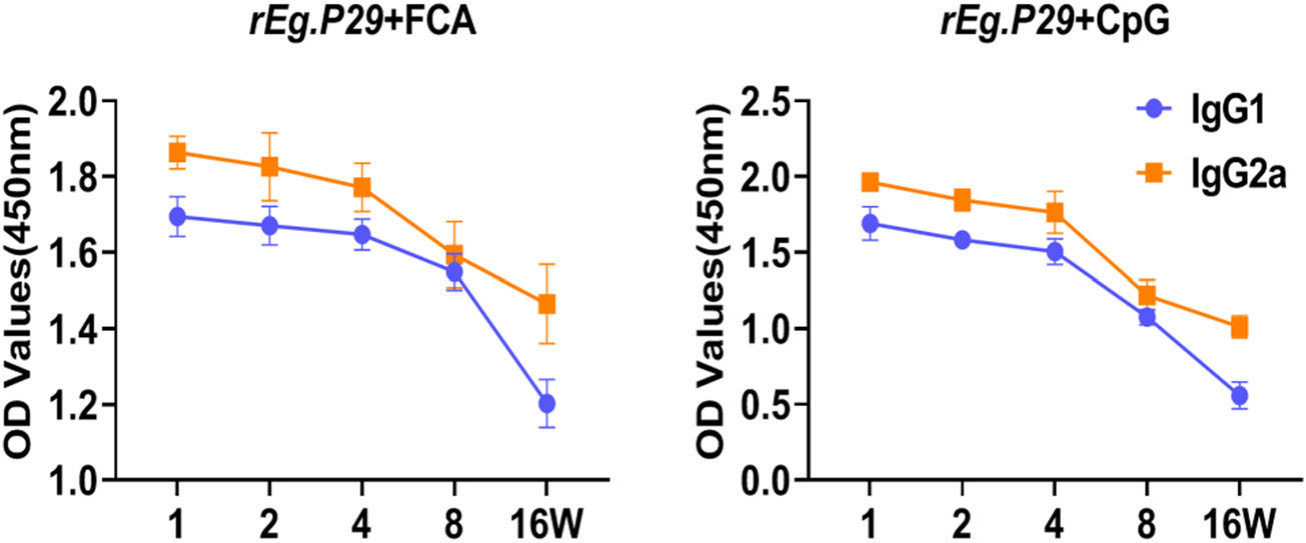
Vaccine‐induced a polarized Th1 response. Anti‐*rEg.P29* specific antibodies were detected by ELISA. IgG1 and IgG2a in serum from immunized mice.****p < .0001, ***p < .001, **p < .01, ns, not significant, *p* > .05

Characterization of IgG isotype responses in *rEg.P29* + FCA and *rEg.P29* + CpG group showed the induction of both IgG1 and IgG2a antibody responses, with the IgG2a isotype showing a comparatively higher level than IgG1 (Figure 2).

### 3.4 Screening for the immunodominant linear B‐cell epitope peptides

**Figure 3:**
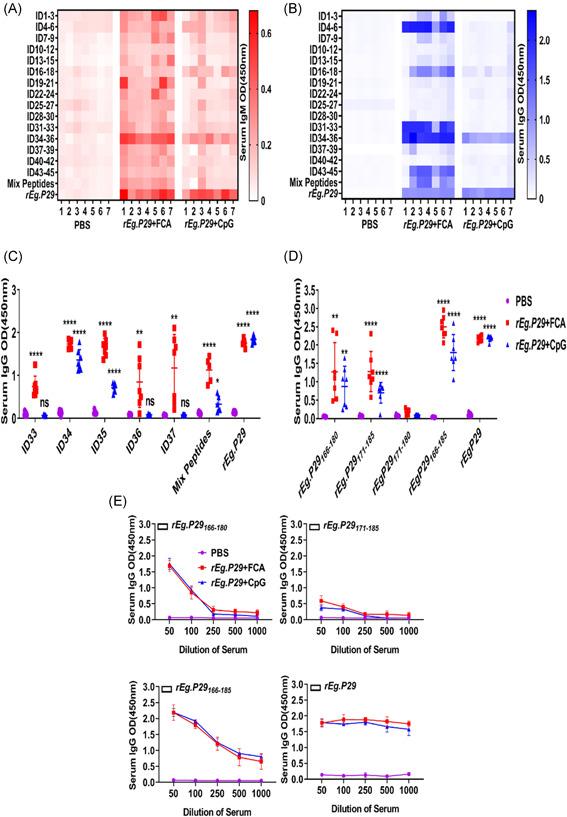
Identification of the core epitope of the immunodominant epitope peptide. ELISA detection of B‐cell epitope peptides of *rEg.P29*. (A, B) To determine the immunodominant epitope peptide of *rEg.P29*, ELISA plate was coated with synthetic peptides or *rEg.P29*. Then, serum samples from C57 mice that were immunized with PBS, *rEg.P29* + FCA, or *rEg.P29* + CpG were detected. The absorbance was read at 450 nm. (C) ELISA plate was coated with ID33‐ID37, mix peptides (ID1–ID45), or *rEg.P29*, specific IgG antibody was detected. (D) To determine the core epitope of immunodominant epitope peptide *rEg.P29*, ELISA plate was coated with four truncated or extended peptides. Then, serum samples from PBS, *rEg.P29* + FCA, or *rEg.P29* + CpG were detected.(E) Titers of IgG in sera from C57.****p < .0001, ***p < .001, **p < .01, ns, not significant, *p* > .05.

The sera of mice immunized with *rEg.P29* and HRP‐labeled goat anti‐mouse IgM and IgG were used as antibodies to detect the interaction between the antigen peptide (Table S1) and the serum. The dominant epitopes were selected, as shown in Figure 3A,B. Compared to the control group, the synthesized antigen peptide (Table S1) could induce ELISA‐specific reactions with the immune serum. ID34‐ID36 IgG and IgM levels were significantly higher than those of the other antigenic peptides in both immune groups.

We further assessed IgG specific for ID33, ID34, ID35, ID36, and ID37 using ELISA. All of these epitopes were detected by peptide‐specific IgG in the *rEg.P29* + FCA group, but only anti‐ID34‐ and anti‐ID35‐specific IgG were significantly higher than other antigenic peptides in both immune groups compared to the control group (*p* < .0001) (Figure 3C). To identify the core epitopes of the immunodominant peptide, we synthesized ID34 (*rEg.P29*_166–180_), ID35 (*rEg.P29*_171–185_), and their overlap (*rEg.P29*_171–180_ and *rEg.P29*_166–185_), as shown in Table 1. The ELISA plate was coated with these four peptides (10 μg/ml, respectively) and the positive control *rEg.P29*. Results indicated that the strongest IgG antibody reactivity was concentrated on a major immunodominant peptide, *rEg.P29*_166–185_ (LKNAKTAEQKAKWEAEVRKD), of *rEg.P29* (*p* < .001, Figure 3D). In addition, anti‐*reg*.P29_166–185_ anti‐*rEg.P29*_*166‐185*_IgG showed comparatively higher titers, as determined among those peptides by endpoint titration. As expected, anti‐*rEg.P29* IgG maintained higher titers than anti‐*rEg.P29*_166–185_ IgG (Figure 3E).

**Table 1:** Amino acid sequence of dominant peptides

SEQ ID	Location	Purity (%)	Sequence (N‐C)	Number
ID34	*rEg.P29*_166–180_	>98%	LKNAKTAEQKAKWEA	15
ID35	*rEg.P29*_171–185_	>98%	TAEQKAKWEAEVRKD	15
	*rEg.P29*_171–180_	>98%	TAEQKAKWEA	10
	*rEg.P29*_166–185_	>98%	LKNAKTAEQKAKWEAEVRKD	20

### 3.5 Detection of antibody subtypes of B‐cell epitope core sequences

**Figure 4:**
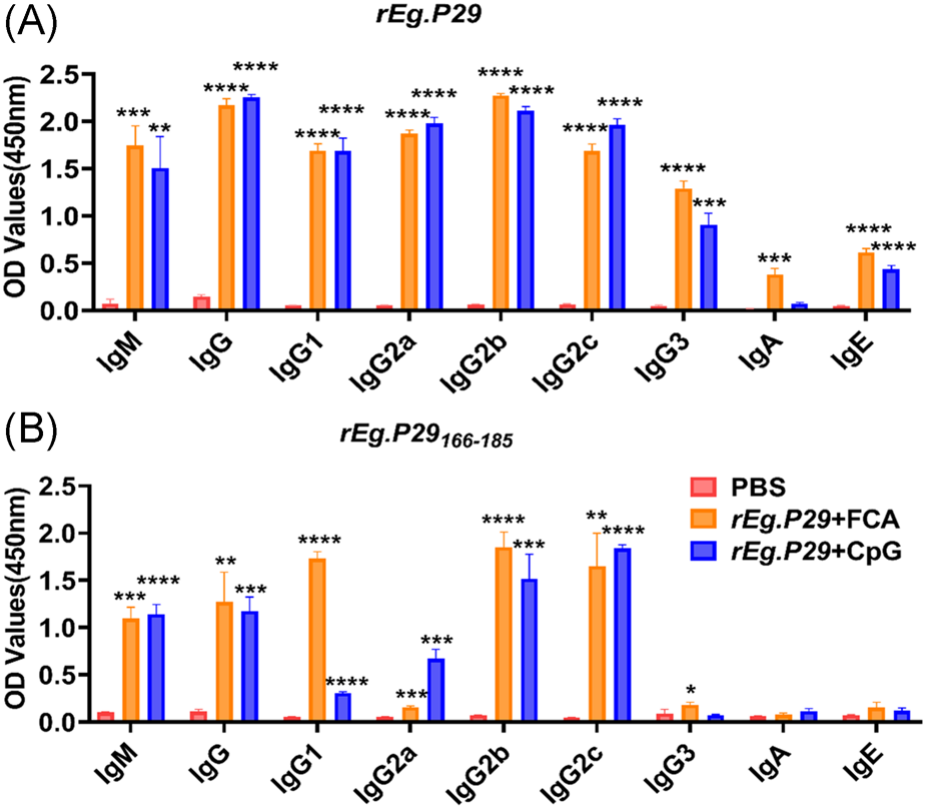
Anti‐*rEg.P29* or core peptide specific antibodies. (A) ELISA plate was coated with *rEg.P29*. Then, serum samples from C57 mice that were immunized with PBS, *rEg.P29* + FCA, or *rEg.P29* + CpG were detected. (B) ELISA plate was coated with *rEg.P29*_166–185_. Then, serum samples from C57 mice that were immunized with PBS, *rEg.P29* + FCA, or *rEg.P29* + CpG were detected.****p < .0001, ***p < .001, **p < .01, ns, not significant, *p* > .05

The ELISA plate was coated with *rEg.P29* and *rEg.P29*_166–185_ to detect antibody subtypes of B‐cell epitope core sequences. Results showed that anti‐*rEg.P29*_166–185_‐specific antibodies lack epitopes against IgA, IgE, and IgG3, compared to anti‐*rEg.P29*‐specific antibodies (Figure 4A,B).

### 3.6 *rEg.P29*_166–185_ and *rEg.P29* promote B‐cell activation and proliferation

**Figure 5:**
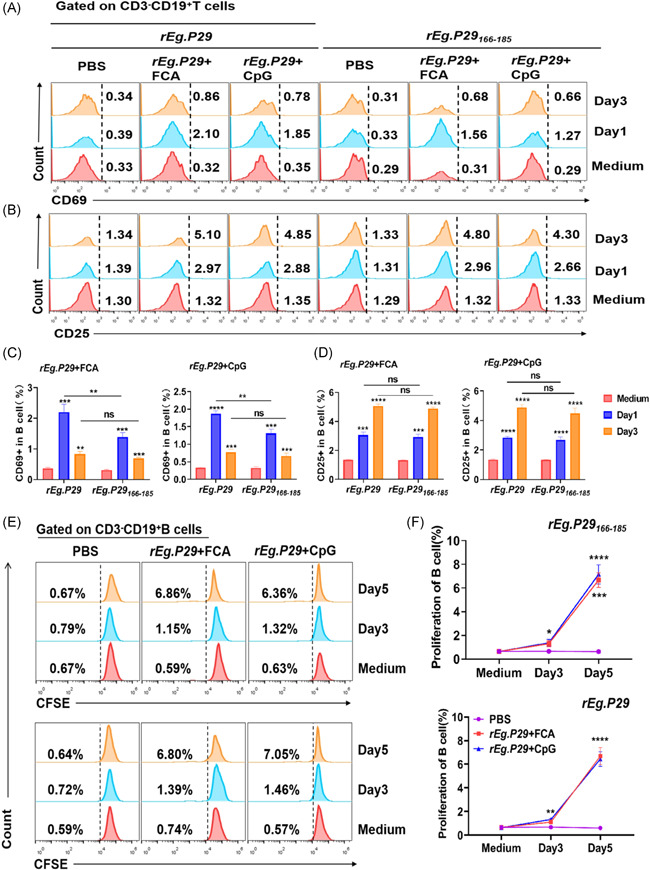
*rEg.P29*_166–185_ and *rEg.P29* promoted specific B‐cell activation and proliferation. Mice were primed and boosted with PBS, *rEg.P29* + FCA, or *rEg.P29* + CpG and killed at Week 2 after boosting. Mononuclear cells from the spleen were isolated. (A–D) Cells were stimulated with *rEg.P29*_166–185_, *rEg.P29,* and medium for 1 and 3 days. The cells were harvested and stained with fluorochrome‐conjugated monoclonal antibodies for CD69 and CD25. Data were collected by flow cytometry and analyzed using FlowJo. Representative dot plots in (A) show CD69 expression in B cells, (B) show CD25 expression in B cells. Frequencies of CD69 expression in B cells from *rEg.P29* + FCA and *rEg.P29* + CpG groups are shown in (C). Frequencies of CD25 expression in B cells from *rEg.P29* + FCA and *rEg.P29* + CpG groups are shown in (D). Cells were used for CFSE labeling and stimulated with *rEg.P29*_166–185_, *rEg.P29,* and medium for 3 and 5 days. The cells were harvested and stained with fluorochrome‐conjugated monoclonal antibodies for CD3 and CD19. Data were collected by flow cytometry and analyzed using FlowJo. Representative dot plots in (E) show the identification of proliferative B cells. Frequencies of proliferative B cells from PBS, *rEg.P29* + FCA, and *rEg.P29* + CpG groups are shown in (F). Data from five mice, ****p < .0001, ***p < .001, **p < .01, ns, not significant, *p* > .05

The expression of CD69 and CD25 was assessed using flow cytometry to study the kinetics of cell activation. Results showed that the expression of CD69 and CD25 from day one was elevated (Figure 5A–D). Gating is shown in Figure S1. Increased CD69 expression in B cells after treatment with *rEg.P29* or *rEg.P29*_166–185_ was significantly different from that in cells cultured without *rEg.P29* or *rEg.P29*_86–100_ (medium) on Day 1 (*p* < .001, *p* < .0001; *p* < .001, *p* < .001). Meanwhile, CD69 expression in B cells decreased on Day 3 (*p* < .01, *p* < .001; *p* < .001, *p* < .001). Additionally, CD25‐expressing cells treated with *rEg.P29* or *rEg.P29*_166–185_ were significantly elevated on Day 3 compared to those not treated with *rEg.P29* or *rEg.P29*_166–185_ (all *p* < .0001, *p* < .0001; *p* < .0001, *p* < .0001). These results suggest that most *rEg.P29*‐specific B cells had already been activated from Day 1. It is worth noting that *rEg.P29*_166–185_ induced slight increase in CD69 than the corresponding increase for *rEg.P29* (*p* < .01, *p* > .05; *p* < .01, *p* > .05); moreover, there was no difference in the magnitude of CD25 elevation (*p* > .05, *p* > .05; *p* > .05, *p* > .05).

Next, we performed CFSE labeling to estimate cell division (Figure 5E,F). After culturing with *rEg.P29* or *rEg.P29*_166–185_, the CFSE intensity of a portion of B cells was decreased at Day 3 (*p* < .01, *p* < .01; *p* < .05, *p* < .05) and further decreased at Day 5 (*p* < .0001, *p* < .0001; *p* < .0001, *p* < .001) compared to the control group.

### 3.7 *rEg.P29*_166–185_ was relatively conserved in different subtypes of P29 sequences

Here, we analyzed the *rEg.P29* sequences of different genotypes of *E. granulosus* and the corresponding regions of the dominant peptides. As shown in Figure S3, the dominant peptide (*rEg.P29*_166–185_) was completely conserved in the P29 sequences of different subtypes.

## 4 DISCUSSION

With the improvement in living standards and sanitary conditions, the incidence rate of *E. granulosus* has been declining annually.^4^ However, it is still a serious public health problem in pastoral areas. New disciplines, such as bioinformatics, immunoinformatics, and rational vaccine design, have been applied in recent years.^19^, ^20^ Among them, the design of a new vaccine composed of multiple epitopes of a single antigen has become a new method to enhance the host's protective immune response at humoral and cellular level. The epitope, also known as the antigenic determinant, is a chemical group in the antigen molecule that determines antigen specificity.^21^ Epitope vaccines are based on the characteristics of the amino acid sequence of antigenic epitopes. They have become a new direction invaccine research because of their highly effective immune protection, safety, and stability.^22^, ^23^

In 2013, Esmaelizad et al.^24^ synthesized a multi‐T‐cell epitope antigen based on five proteins: EgGST, EgA31, Eg95, Eg14‐3, and EgTrp. The antigen‐stimulated mouse spleen cells to produce IFN‐γ and obtained 99.6% protective efficacy in a mouse model. Simultaneously, multiepitope vaccines are also used in other fields.^25^, ^26^, ^27^ The recombinant Bacillus Calmette–Guérin (BCG) vaccine (rBCG) constructed by Mohamud et al.^28^ included two peptides, rBCG018 and rBCG032, that exhibited a stronger ability to induce cellular and humoral immune responses than BCG. Compared with traditional vaccines, synthetic peptide vaccines are easily prepared, have stable structure, and do not risk infection; thus, they are used for novel vaccine design at present.

Our previous studies have demonstrated that *rEg.P29* has a favorable protective effect in sheep and mouse models. However, the B‐cell epitopes of *rEg.P29* remain unclear. Identification of the B‐cell epitopes of *rEg.P29* contributes not only in elucidating the mechanism underlying the B‐cell immune process of *E. granulosus* but also to the development of more effective epitope vaccine candidates. Screening epitopes with high immunogenicity is used for epitope development. Here, after the primer and booster doses of *rEg.P29*, the specific antibody levels peaked at Week 2. Immunized mice expressed IgM, IgG, IgG2a, and IgG2b, and few expressed IgA and IgE. Antibody production was maintained for at least 16 weeks, except for IgA and IgE. Studies have shown that IgG, IgG2a, and IgG2b may participate in the protective immune response induced by the vaccine, and IgG1 may be related to susceptibility to hydatidosis. Meanwhile, IgG3 may be associated with the formation of a local inflammatory response early in the host.^29^ Th2‐related antibodies and cytokines mainly promote the escape of parasites and establish immune tolerance, while Th1 related antibodies and cytokines are closely related to anti‐infection.^30^, ^31^ Serological antibody tests showed that the immunized mice had a predominantly IgG and IgG2a antibody response against *rEg.P29*, followed by IgG1. Moreover*rEg.P29* immunization induced a predominantly Th1 immune response. Our results showed that the dominant epitope lacks anti‐*rEg.P29*_166–185_ specific IgA, IgE, and IgG3 antibodies compared to anti‐*rEg.P29* specific antibodies. These results suggest that *rEg.P29*_166–185_ may only induce protective immunity while eliminating allergic reactions.^32^, ^33^ However, this difference may be caused by the adjuvant or the weak immunogenicity of the peptide, suggesting that multiple epitopes or vectors should be combined to improve the immunogenicity of the peptide. Furthermore, lymphocyte activation and proliferation levels are indicators that reflect the body's immune function. Here, we screened *rEg.P29*_166–185_, for its ability to activate and proliferate B cells using flow cytometry. Results showed that *rEg.P29*_166–185_ and *rEg.P29* promote B‐cell activation and proliferation, highlighting that specific B cells can rapidly activate and proliferate in response to infection.

In addition, sequence alignment of the dominant epitope with the P29 protein of different isolates of *E. granulosus* revealed that *rEg.P29*_166–185_ was highly conserved, suggesting its application as a broad‐spectrum vaccine.^34^

Two adjuvants were used in the present study. CpG adjuvant is an oligodeoxynucleotide containing a CpG sequence with strong immune‐activating properties; it was reported to show few side effects and can be used in humans.^35^ Our results showed that both CpG and Freund's adjuvant enhanced the humoral immune response. However, Freund's adjuvant induces severe local necrotic ulcers and is considered to be toxic for human use. Considering the translational application of the vaccine, we used CpG adjuvant in subsequent experiments. Nonetheless, studies have shown that adjuvants alone do not induce specific antibody responses,^36^ as shown in Figure S2.

This study has some limitations. Our study did not validate the immunoprotective effect after immunization of the dominant epitope. Further studies are warranted.

## 5 CONCLUSION

In conclusion, our studies show that *rEg.P29* immunization can induce a strong and sustained antibody response, and *rEg.P29*_166–185_ can act as its dominant B‐cell epitope. Furthermore, *rEg.P29* and *rEg.P29*_166–185_ stimulation in vitro can promote B‐cell activation and proliferation.

## ETHICS STATEMENT

All mouse experiments were given permission by the Ningxia Medical University Institutional Review Committee (Permit number: SCXK2016‐0006) and carried out in strict accordance with national and institutional guidelines. The data obtained met the appropriate ethical requirements.

## AUTHOR CONTRIBUTIONS

Wei Zhao and Changyou Wu were in charge of the project design; Yongxue Lv and Shasha Li were responsible for conducting the experiments, the data analysis, and paper writing; Tingrui Zhang and Jihui Yang were responsible for guiding Flow analysis in the experiment; Jia Tao was responsible for the design of relevant peptides in the experiment; Liangliang Chang and Yazhou Zhu were responsible for animal feeding; Yongxue Lv was in charge of the revision; Wei Zhao and Changyou Wu were responsible for the experimental guidance; all authors read and approved the final manuscript.

## Supporting information

Supplementary information.

## Data Availability

Contact the corresponding author for data and material requests.
