# Supplementary material for: Identification of B‐cell dominant epitopes in the recombinant protein P29 from Echinococcus granulosus
Source: Immun Inflamm Dis. 2022 Apr 19;10(5):e611. doi: 10.1002/iid3.611 (PMC9017632; doi:10.1002/iid3.611)
Supplement: Supplementary file 1 — Supplementary information. [file IID3-10-e611-s001.docx]

**
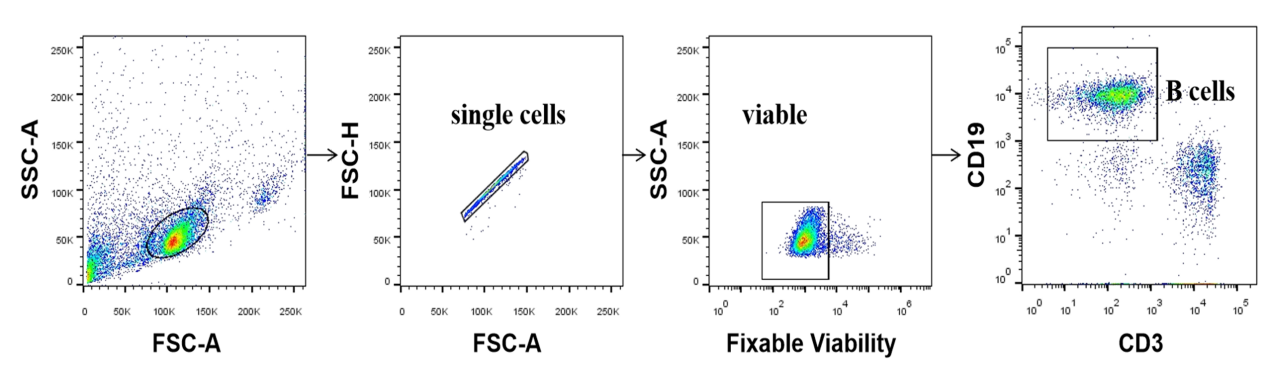
**

**Supplementary Figure 1.** After gating on lymphocytes,single cells, live cells, CD3^-^CD19^+^ B cells were divided based on CD3 and CD19 antibodies.


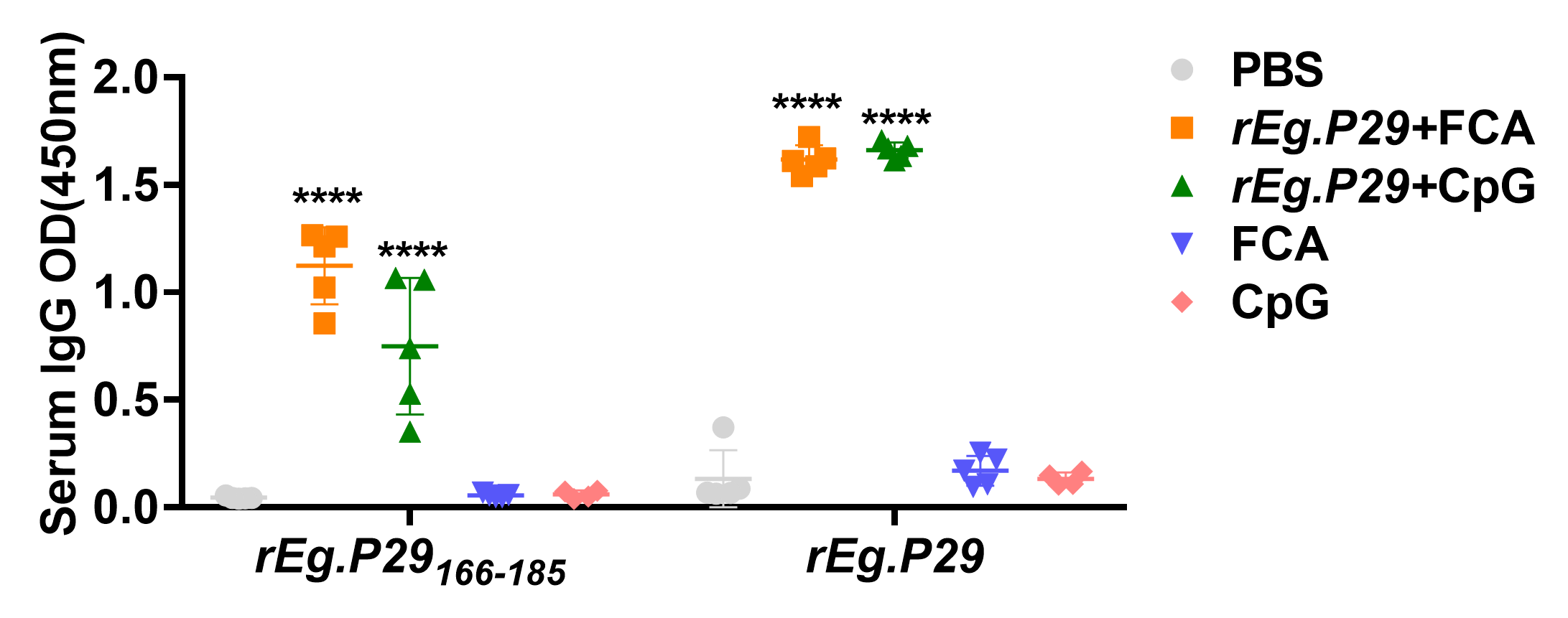


**Supplementary Figure 2.** **Adjuvants did not induce specific antibody production.**

Anti-*rEg.P29* specific antibodies were detected by ELISA.IgG antibody in serum from immunized mice.*****P* < 0.0001, ****P* < 0.001, ***P* < 0.01, ns= not significant, *P* > 0.05.


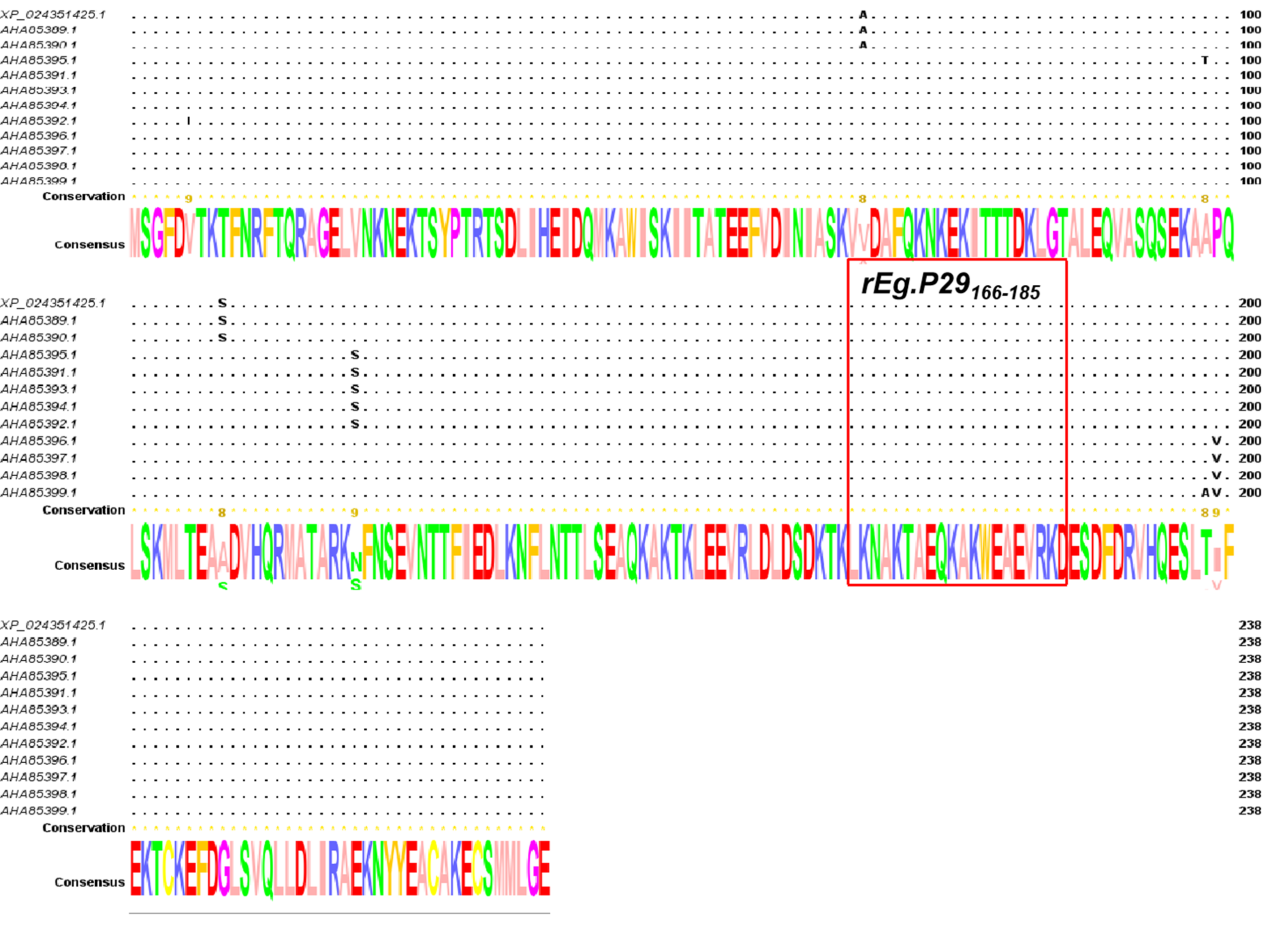


**Supplementary Figure 3. Amino acid sequence alignment.**Homology was analyzed using BLAST and UniProt.

**Supplementary Table1**

Amino acid sequences of overlapping peptides

| **No.** |  | **Sequence** | **number** |
| --- | --- | --- | --- |
| ID1 | *rEg.P29_1-15_* | MSGFDVTKTFNRFTQ | 15 |
| ID2 | *rEg.P29_6-20_* | VTKTFNRFTQRAGEL | 15 |
| ID3 | *rEg.P29_11-25_* | NRFTQRAGELVNKNE | 15 |
| ID4 | *rEg.P29_16-30_* | RAGELVNKNEKTSYP | 15 |
| ID5 | *rEg.P29_21-35_* | VNKNEKTSYPTRTSD | 15 |
| ID6 | *rEg.P29_26-40_* | KTSYPTRTSDLIHEI | 15 |
| ID7 | *rEg.P29_31-45_* | TRTSDLIHEIDQMKA | 15 |
| ID8 | *rEg.P29_36-50_* | LIHEIDQMKAWISKI | 15 |
| ID9 | *rEg.P29_41-55_* | DQMKAWISKIITATE | 15 |
| ID10 | *rEg.P29_46-60_* | WISKIITATEEFVDI | 15 |
| ID11 | *rEg.P29_51-65_* | ITATEEFVDINIASK | 15 |
| ID12 | *rEg.P29_56-70_* | EFVDINIASKVADAF | 15 |
| ID13 | *rEg.P29_61-75_* | NIASKVADAFQKNKE | 15 |
| ID14 | *rEg.P29_66-80_* | VADAFQKNKEKITTT | 15 |
| ID15 | *rEg.P29_71-85_* | QKNKEKITTTDKLGT | 15 |
| ID16 | *rEg.P29_76-90_* | KITTTDKLGTALEQV | 15 |
| ID17 | *rEg.P29_81-95_* | DKLGTALEQVASQSE | 15 |
| ID18 | *rEg.P29_86-100_* | ALEQVASQSEKAAPQ | 15 |
| ID19 | *rEg.P29_91-105_* | ASQSEKAAPQLSKML | 15 |
| ID20 | *rEg.P29_96-110_* | KAAPQLSKMLTEASD | 15 |
| ID21 | *rEg.P29_101-115_* | LSKMLTEASDVHQRM | 15 |
| ID22 | *rEg.P29_106-120_* | TEASDVHQRMATARK | 15 |
| ID23 | *rEg.P29_111-125_* | VHQRMATARKNFNSE | 15 |
| ID24 | *rEg.P29_116-130_* | ATARKNFNSEVNTTF | 15 |
| ID25 | *rEg.P29_121-135_* | NFNSEVNTTFIEDLK | 15 |
| ID26 | *rEg.P29_126-140_* | VNTTFIEDLKNFLNT | 15 |
| ID27 | *rEg.P29_131-145_* | IEDLKNFLNTTLSEA | 15 |
| ID28 | *rEg.P29_136-150_* | NFLNTTLSEAQKAKT | 15 |
| ID29 | *rEg.P29_141-155_* | TLSEAQKAKTKLEEV | 15 |
| ID30 | *rEg.P29_146-160_* | QKAKTKLEEVRLDLD | 15 |
| ID31 | *rEg.P29_151-165_* | KLEEVRLDLDSDKTK | 15 |
| ID32 | *rEg.P29_156-170_* | RLDLDSDKTKLKNAK | 15 |
| ID33 | *rEg.P29_161-175_* | SDKTKLKNAKTAEQK | 15 |
| ID34 | *rEg.P29_166-180_* | LKNAKTAEQKAKWEA | 15 |
| ID35 | *rEg.P29_171-185_* | TAEQKAKWEAEVRKD | 15 |
| ID36 | *rEg.P29_176-190_* | AKWEAEVRKDESDFD | 15 |
| ID37 | *rEg.P29_181-195_* | EVRKDESDFDRVHQE | 15 |
| ID38 | *rEg.P29_186-200_* | ESDFDRVHQESLTIF | 15 |
| ID39 | *rEg.P29_191-205_* | RVHQESLTIFEKTCK | 15 |
| ID40 | *rEg.P29_196-210_* | SLTIFEKTCKEFDGL | 15 |
| ID41 | *rEg.P29_201-215_* | EKTCKEFDGLSVQLL | 15 |
| ID42 | *rEg.P29_206-221_* | EFDGLSVQLLDLIRA | 15 |
| ID43 | *rEg.P29_211-226_* | SVQLLDLIRAEKNYY | 15 |
| ID44 | *rEg.P29_216-231_* | DLIRAEKNYYEACAK | 15 |
| ID45 | *rEg.P29_221-236_* | EKNYYEACAKECSMM | 15 |
